# Successful containment of COVID-19: the WHO-Report on the COVID-19 outbreak in China

**DOI:** 10.1007/s15010-020-01409-4

**Published:** 2020-03-17

**Authors:** Bernd Salzberger, Thomas Glück, Boris Ehrenstein

**Affiliations:** 1 Department Infection Control and Infectious Disease, University of Regensburg, Regensburg, Germany; 2 Department Internal Medicine, Kreisklinikum Trostberg, Trostberg, Germany; 3 Department Rheumatology, Asklepios Medical Center, Bad Abbach, Germany; 4 Abt. Krankenhaushygiene und Infektiologie, Universitätsklinikum Regensburg, Franz-Josef-Strauss-Allee 11, 93053 Regensburg, Germany

SARS-CoV-2 is a new Coronavirus, with first infections detected in humans late in 2019. The emergence of SARS-CoV-2 has led to a large outbreak in China and is currently causing outbreaks in many countries. The disease spectrum ranges from uncomplicated upper respiratory tract infections to severe viral pneumonia with multiorgan failure and death. It can be transmitted by droplets from asymptomatic or oligosymptomatic patients and possibly through aerosols in health care environments.

The route of transmission and the spectrum of disease (COVID-19) has motivated many researchers to use models of influenza outbreaks or pandemics to forecast outbreaks of SARS-CoV-2 by analogy.

The epicenter of China’s outbreak has been Wuhan and the Hubei province. The Chinese government has restricted travel from and to Hubei province and has implemented a number of measures to contain the outbreak. Meanwhile, the number of new cases per day in China is falling. A WHO mission has visited China and Wuhan to report on the outbreak. They corroborated the outbreak dynamic and case count reported by the Chinese government [2].

## The Chinese success-estimating an upper limit to the attack rate in Hubei province

Intensive public health interventions have been employed, and some experts expect the outbreak to end as early as in April. The bundle of public health interventions has included intensive case and contact tracking, isolation of moderately ill patients in containment centers, social distancing, and shutting down public life of a whole province and many major cities outside Hubei.

Just how effective the outbreak seems to have been contained is astonishing. Publicly available data can be employed to estimate the attack rate of the COVID-19 outbreak in China. There are two datasets with a very different picture of the same epidemic caused by the same virus [3].

1. Data from Hubei (roughly 80% of the China outbreak with a focus on severe cases and high case fatality rate (CFR), currently cumulatively estimated to be around 4%).
2. Data from China outside Hubei province with a probably much better coverage of the whole epidemic due to active case finding.

**Fig. 1:**
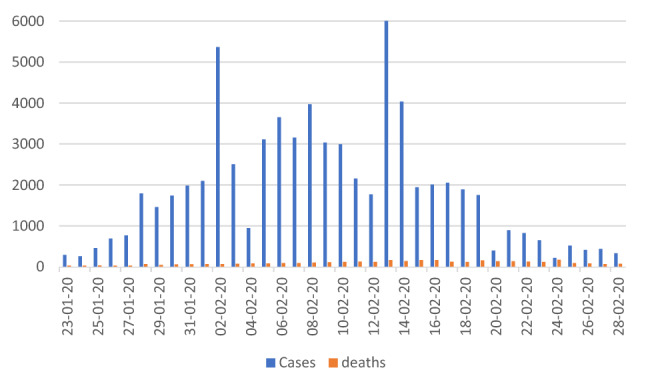
COVID-19 cases/death in China by day. Daily cases and deaths in China, case count for February 13 truncated (change of case definition, 16,119 cases in the part retrospectively reported) (adapted from 3)

**Fig. 2:**
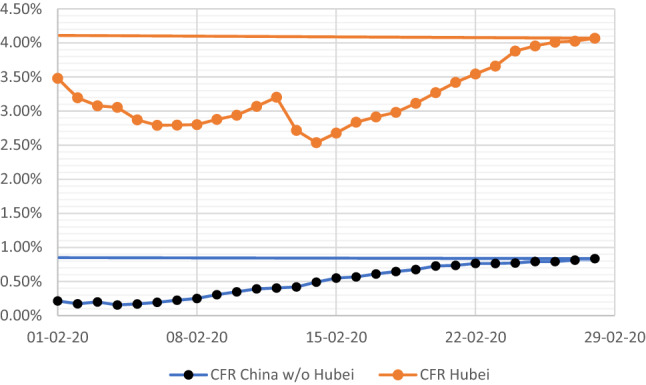
CFR Hubei and China outside Hubei. CFR in Hubei (orange) and in China outside Hubei province (black), calculated from daily cumulative case and deaths numbers. Horizontal lines indicate current status (adapted from 3)

With steadily declining case numbers and numbers of new deaths also declining in the second group, the case fatality rate in China outside Hubei province is stabilizing around 0.8% (Fig. 1). If we put these two datasets parallelly we could cautiously (to be on the safe side for an upper limit estimate) assume, that up to 50% of cases might still be missed outside Hubei province with the consequence of a lower case fatality rate, because severe cases are unlikely to be missed (Fig. 2).

The second cautious assumption would be that the current caseload in Hubei province represents only 2/3 of the final caseload, putting the total number to approximately 100.000 cases (with the current clinical characteristics).

To set this parallelly with the epidemic outside Hubei province, we would have to multiply this case count by 5–10 (five if the data outside Hubei reflect all cases, ten for under reporting of 50%).

Thus, the number of cases in the Hubei outbreak could be estimated as between 500.000–1.000.0000. With a population of 57 million people in Hubei province, the attack rate would be below 2%.

## How is COVID-19 different from Influenza?

This estimate of an upper limit of 2% is considerably lower than previous forecasts and estimates in analogy to influenza pandemics or outbreaks (Table 1). Compared to influenza outbreaks the attack rate and burden of disease in children have been much lower and the secondary household attack rate has also been low (Table 1). This is in sharp contrast to observations of a very rapid spread of the virus in confined situations as prisons or cruise ships and the high rate of healthcare-associated infections. Several hypotheses will have to be explored to answer at least some of the questions: (1) initial estimates of R_0_ might have been biased by clusters of effective transmission (“super-spreaders”), (2) public health measures might be more effective to reduce R_t_ in SARS-CoV-2 than in influenza outbreaks and (3) whether there is a threshold of prevalent cases in the community, which if reached, the epidemic can be effectively contained only with drastic measures.

**Table 1:** Attack rates of pandemic or seasonal influenza and COVID-19 in Hubei province [1, 2]

	Influenza 1957	Influenza 1968	Influenza 2009	Influenza 1977–8	COVID-19, Hubei 2020
Community attack rate confirmed cases	18.5–26.8%	15%	17,5	2.2–31%	< 2%
Secondary household contact attack rate	8.4–23%^a^	20%^a^	4–6%	16%	3–10%

^a^Not laboratory confirmed

## Conclusions and lessons

First, the WHO report is very good news for the people in Hubei province and all health care workers involved.

Second, the success of the interventions demonstrates that strict and rapid response to an emerging epidemic can halt the spread of a new virus.

But there are also some sobering insights looking at the current situation outside China and the messages in the WHO report. China’s success might not be the end of their outbreak. An attack rate as low as 2% could cause a second wave rapidly, because the community level of immunity is still low. Furthermore, the virus has been imported in a large number of countries, which are facing difficult choices regarding public health measures and challenges to their health care system.

The outbreak in Hubei province has shown how much harm a newly emerging respiratory virus can cause. Infections in confined spaces, such as prisons or cruise ships, can rapidly spread, complications can be severe and health care-associated transmission poses a risk for HCWs and other patients. Health care workers from all over China have come to Hubei to help and have been doing excellent and very trying work in treating the large number of patients with a very high number of hospitalized and critically ill patients. Their growing experience in patient care is also reflected in the declining case fatality rate and the declining number of healthcare-associated transmission over the time course of the outbreak. The resulting publications of clinical data will be very helpful for patient care outside Hubei province and as clinicians we will profit immensely from those. But we do not know how the situation in Hubei might have been if the virus had spread early to other metropolitan centers in China. The workload for healthcare workers would have been multiplied, a collapse of the healthcare system would have been possible and the death toll would have been very high. In the light of these consequences, any public health intervention seems to be a better option.

So, despite the good news from China, the work is far from over. Outside China, we face enormous challenges: (1) to effectively contain the current and future outbreaks worldwide, and (2) to treat infected patients effectively and safely. Looking at the Chinese experience, we hope that public health measures outside China will be as rapid and effective as in China. We should implement those before reaching a critical threshold of infections.
